# The Combined Repetitive Oligopeptides of *Clostridium difficile* Toxin A Counteract Premature Cleavage of the Glucosyl-Transferase Domain by Stabilizing Protein Conformation

**DOI:** 10.3390/toxins6072162

**Published:** 2014-07-22

**Authors:** Alexandra Olling, Corinna Hüls, Sebastian Goy, Mirco Müller, Simon Krooss, Isa Rudolf, Helma Tatge, Ralf Gerhard

**Affiliations:** 1 Institute of Toxicology, Hannover Medical School, Carl-Neuberg-Str. 1, 30625 Hannover, Germany; E-Mails: olling.alexandra@mh-hannover.de (A.O.); corri.H88@gmx.de (C.H.); goy.sebastian@mh-hannover.de (S.G.); simonkrooss@icloud.com (S.K.); rudolf.isa@mh-hannover.de (I.R.); tatge.helma@mh-hannover.de (H.T.); 2 Institute for Biophysical Chemistry, Hannover Medical School, Carl-Neuberg-Str. 1, 30625 Hannover, Germany; E-Mail: mirco_mueller@yahoo.de

**Keywords:** *Clostridium difficile* toxin, domain interaction, autoprocessing, cytotoxicity, microscale thermophoresis

## Abstract

Toxin A (TcdA) and B (TcdB) from *Clostridium difficile* enter host cells by receptor-mediated endocytosis. A prerequisite for proper toxin action is the intracellular release of the glucosyltransferase domain by an inherent cysteine protease, which is allosterically activated by inositol hexaphosphate (IP_6_). We found that in *in vitro* assays, the *C*-terminally-truncated TcdA^1–1065^ was more efficient at IP_6_-induced cleavage compared with full-length TcdA. We hypothesized that the *C*-terminally-located combined repetitive oligopeptides (CROPs) interact with the *N*-terminal part of the toxin, thereby preventing autoproteolysis. Glutathione-*S*-transferase (GST) pull-down assays and microscale thermophoresis confirmed binding between the CROPs and the glucosyltransferase (TcdA^1–542^) or intermediate (TcdA^1102–1847^) domain of TcdA, respectively. This interaction between the *N*- and *C*-terminus was not found for TcdB. Functional assays revealed that TcdB was more susceptible to inactivation by extracellular IP_6_-induced cleavage. *In vitro* autoprocessing and inactivation of TcdA, however, significantly increased, either by acidification of the surrounding milieu or following exchange of its CROP domain by the homologous CROP domain of TcdB. Thus, TcdA CROPs contribute to the stabilization and protection of toxin conformation in addition to function as the main receptor binding domain.

## 1. Introduction

Toxins A (TcdA) and B (TcdB) are the main virulence factors of *Clostridium difficile* and predominantly responsible for *C. difficile*-induced diseases, ranging from mild diarrhea to fulminant pseudomembranous colitis and toxic megacolon [1,2]. Following secretion into the gut, the toxins enter their target cells via receptor-mediated endocytosis after binding to the cell surface, at least by the *C*-terminally-located combined repetitive oligopeptides (CROPs) [3,4,5]. Endosomal acidification triggers conformational changes of TcdA and TcdB, resulting in vesicle membrane insertion and translocation of the *N-*terminus into the cytosolic compartment [6]. The outer *N*-terminal subunit harbors the glucosyltransferase (GT-) domain, which inactivates small Rho GTPases by mono-glucosylation. This leads to disruption of the actin cytoskeleton and, consequently, cell rounding [7]. As a prerequisite for substrate modification, the GT-domain is cleaved off and released into the cytosol by the action of the adjacent cysteine protease domain [8]. The function of the inherent protease is allosterically activated by reducing conditions and the binding of cytosolic inositol hexakisphosphate (IP_6_) [9]. The importance of autocatalytic processing with regard to toxin action becomes evident by TcdA A^541^G^542^A^543^, a cleavage-resistant mutant, which results in about a 75-fold reduction of cytotoxic potency compared to wild-type TcdA. Instead, extracellular cleavage prevents toxin-mediated cellular effects [10]. Although structural and functional elucidation of the individual toxin domains allowed insights into the multistep process of toxin uptake and toxicity, the interaction of the functional domains in the context of the full-length toxin is rarely investigated. Besides a low resolution analysis of TcdB-structure obtained by small-angle X-ray scattering (SAXS) [11], Pruitt and co-workers presented a structural model of full-length TcdA based on negative stain electron microscopy followed by 3D-reconstruction and mapping of the known functional domains [12]. These analyses revealed a closely-packed conformation of TcdA at neutral pH, assuming intramolecular contacts between the individual domains. Under acidic conditions, the molecule takes on a more elongated shape, reflecting toxin unfolding, necessary during endosomal translocation.

In order to understand the interaction of the functional toxin domains, which leads to a conformation that enables the protection of enzymatic domains and molecule flexibility, we analyzed a putative binding between the CROPs and other domains of the toxins and evaluated the functional consequences by *in vitro* cleavage assays. Here, we report that the CROPs of TcdA tightly interact with the residual molecule, which prevents premature autoproteolysis and, thus, inactivation of the toxin. In addition to the commonly accepted function in receptor binding, we therefore propose that the CROPs, at least of TcdA, play an important role in the conformation stability and protection of the toxin.

## 2. Results and Discussion

### 2.1. Efficiency of pH-Dependent Autoprocessing Differs between Full-Length TcdA and C-Terminally-Truncated Toxin Fragments

**Figure 1:**
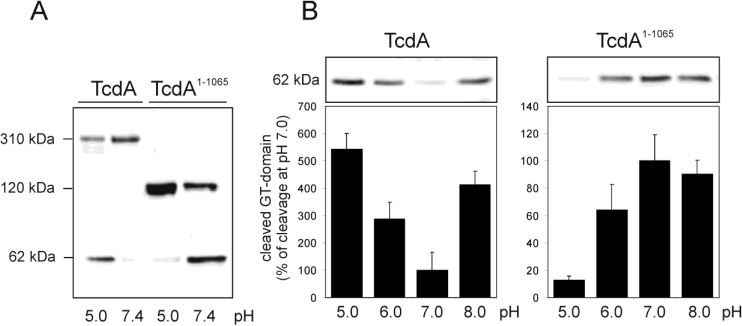
pH-dependent efficacy of autoproteolytic cleavage of toxin A (TcdA) and TcdA fragments. (**A**) Inositol-hexakisphosphate (IP_6_)-induced cleavage of TcdA (308 kDa) and TcdA^1–1065^ (120 kDa) at pH 5.0 and pH 7.4. The TcdA-specific immunoblot illustrates unprocessed toxins and the cleaved glucosyltransferase domain (62 kDa); (**B**) Western blot (**upper panel**) and densitometric analyses (**lower panel**) reflecting the cleaved GT-domain of TcdA and TcdA^1–1065^ in dependence of pH. Data are presented as means ± SD (*n* = 3).

Intracellular autoproteolytic processing of TcdA and TcdB was emulated in a cell-free system by incubating the toxins in the presence of inositol hexakisphosphate (IP_6_) and dithiothreitol (DTT). IP_6_/DTT-incubation of fragment TcdA^1–1065^, which lacks the intermediate and the CROP region of TcdA, resulted in about a 50% cleaved glucosyltransferase (GT-) domain at pH 7.4, whereas IP_6_-induced cleavage of the full-length toxin was completely ineffective (Figure 1A). Interestingly, the reduction of pH to an acidic milieu stimulated autoprocessing of TcdA dramatically, as shown by western blot analysis targeting the 62-kDa GT-domain. We therefore systematically compared full-length TcdA and fragment TcdA^1–1065^ with regard to the pH-dependency of cleavage (Figure 1B). Densitometric evaluation of western blots detecting the cleaved GT-domain illustrates that autoproteolytic processing of *C*-terminally-truncated TcdA^1–1065^ is more efficient the more the surrounding milieu gets neutralized (right panel). Opposite results were obtained with full-length TcdA, whose cleavage efficacy continuously decreases with neutralization from pH 5 to pH 7 by a factor of five (left panel).

**Figure 2:**
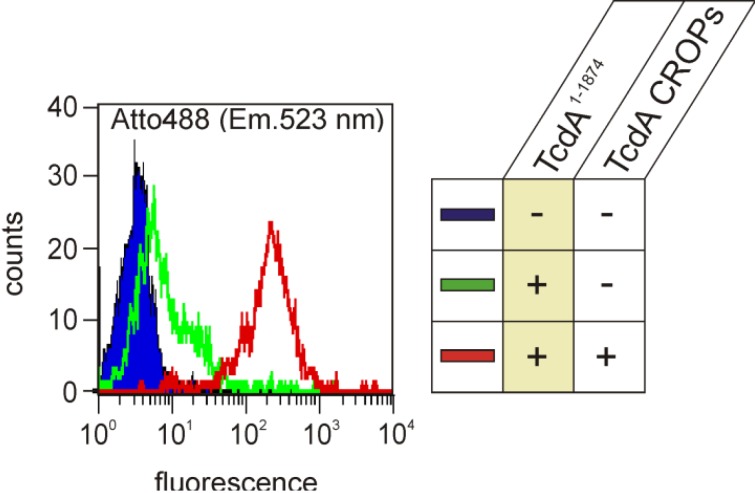
TcdA located combined repetitive oligopeptides (CROPs) facilitate the binding of TcdA^1–1874^ to HT29 cells. Flow cytometry analysis shows the binding of fluorescently (Atto488)-labeled TcdA^1–1874^ to HT29 cells (green curve) and to HT29 cells preloaded with TcdA CROPs (red curve). The blue peak with the black line represents the autofluorescence of HT29 cells in the absence of labeled TcdA^1–1874^. The excitation and emission wavelengths of the fluorophore are 501 nm and 523 nm, respectively, setting the band-pass width to 10 nm.

Assuming that the cleavage activity of partially-denaturated toxin at pH 5 is solely determined by the cysteine protease activity, a cleavage efficiency of roughly 10% can be estimated for TcdA, as well as for TcdA^1–1065^. From this, it can be extrapolated that at pH 7, about 2% of the holotoxin and about 70% of TcdA^1–1065^ will be cleaved. These observations indicate that the intramolecular structures of the full-length toxin impede autoproteolytic cleavage at neutral pH, however, which are abrogated either under acidic conditions or in the case of toxin fragments lacking the *C*-terminus, respectively. The latter hypothesis indicates that the *C*-terminally-located CROPs are essentially involved in the maintenance of a closed, cleavage-protecting conformation and may even form an intramolecular bonding. Further flow cytometry experiments using HT-29 cells supported the assumption of the CROPs interacting with the rest of the toxin. Binding of fluorescently-labeled TcdA^1–1874^, a mutant solely lacking the CROPs, was monitored and illustrated by a right shift in fluorescence (green curve) in Figure 2. To our surprise, previous saturation of the cell surface with the isolated TcdA CROP domain (amino acids 1875–2710) dramatically increased the intensity of fluorescence emitted from subsequent bound TcdA^1–1874^ by a factor of 50 (red curve).

**Figure 3:**
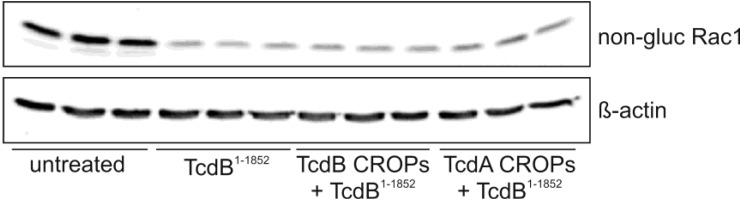
TcdB CROPs do not facilitate the uptake of TcdB^1–1852^. Western blots showing the level of non-glucosylated Rac1 (**upper panel**) as a marker for the intracellular action of TcdB in HT-29 cells in triplicate. β-Actin (**lower panel**) served as the loading control. Rac1 glucosylation by CROP-truncated TcdB^1–1852^ was not altered in dependence of the pre-incubation of cells with the CROPs of TcdA or TcdB, respectively.

In an earlier study, we found that enhanced binding of truncated TcdA^1–1874^ to HT29 cells pre-incubated with TcdA CROPs was associated with a faster internalization process, as determined by glucosylated Rac1 [4]. Interestingly, pre-incubation of HT29 cells with TcdA- or TcdB CROPs did not enhance the internalization of the homologous TcdB^1–1852^ (Figure 3), which also indicates a lack of interaction of TcdB CROPS with the residual protein.

### 2.2. The TcdA CROPs, but Not the CROPs of TcdB, Interact with the Rest of the Respective Toxin

**Figure 4:**
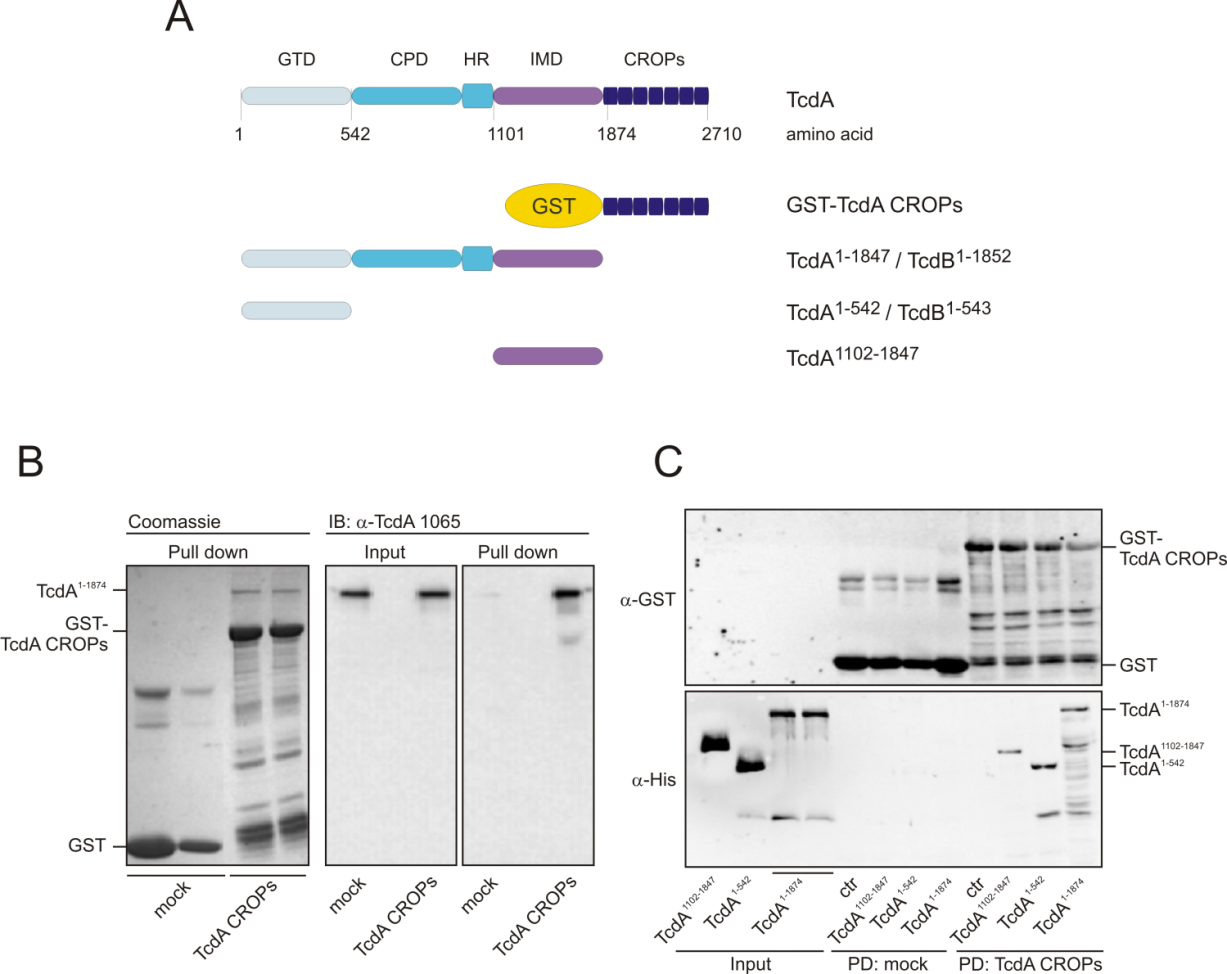
TcdA CROPs interact with TcdA^1–1874^. (**A**) TcdA/TcdB are multi-domain toxins with a glucosyltransferase domain (GTD), cysteine protease domain (CPD), a hydrophobic region (HR), an intermediate domain (IMD) and the CROPs; (**B**) Glutathione-*S*-transferase (GST) (mock) or GST-TcdA CROPs were coupled to GSH beads and used as bait to precipitate CROP-truncated TcdA^1–1874^. Coomassie staining proves equal applied amounts of GST and GST-TcdA CROPs (**left panel**). Immunoblot with α-TcdA^1–1065^ shows the input and precipitation of truncated TcdA (**right panels**); (**C**) Binding of intermediate TcdA^1102–1847^, the glucosyltransferase domain, TcdA^1–542^, and the CROP-deletion mutant, TcdA^1–1874^, to GST-fused TcdA CROPs was analyzed. Immunoblot targeting GST reflects equal amounts of bait (**upper panel**); immunoblot against the Penta-His tag displays the input and bound fraction of the *N*-terminal toxin fragments (**lower panel**).

In order to analyze a potential interaction between the *C*-terminal CROP domain and the *N*-terminal part of the toxin, we performed glutathione-*S*-transferase (GST) pull-down assays using GST-fused CROPs as bait. Therefore, we coupled either GST or GST-CROPs to GSH beads, followed by incubation with the TcdA CROP deletion mutant, TcdA^1–1874^. Toxin fragments used for pull-down experiments are shown in Figure 4a.

**Figure 5:**
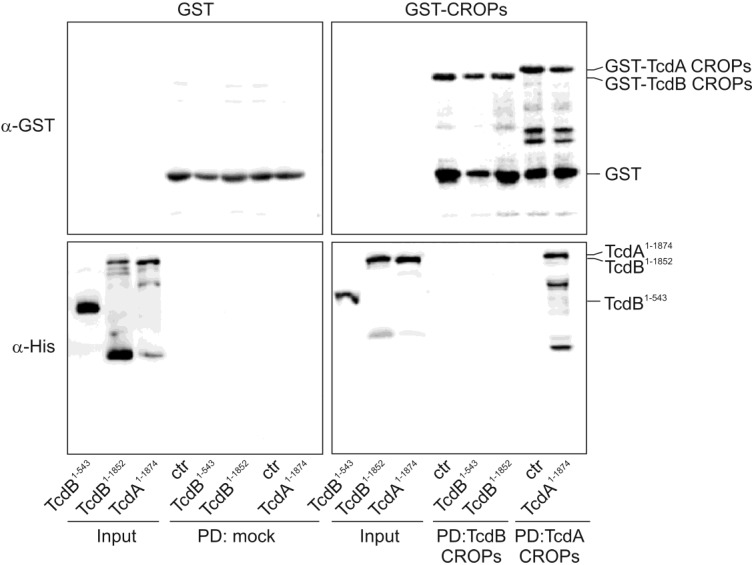
TcdB CROPs do not associate with TcdB^1–1852^. Immunoblots showing pull-downs of GST-fused TcdB CROPs and TcdB fragments (TcdB^1–543^ and TcdB^1–185^). Precipitation of TcdA^1–1874^ by GST-TcdA CROPs served as the positive control.

Immunoblot analyses with an antibody targeting the *N*-terminal part of TcdA (α-TcdA^1–1065^) revealed that TcdA^1–1874^ was specifically precipitated by the CROPs of TcdA, whereas almost no signal was detected when applying GST-coupled beads (mock) as negative bait (Figure 4B). Coomassie staining proved equal amounts of input. However, the question was which domain of TcdA interacts with the CROPs. Referring to the structural model of Pruitt and co-workers, which is based on negative stain electron microscopy followed by 3D reconstruction [12,13], we applied the *N*-terminal GT-domain (TcdA^1–542^) or the poorly-characterized intermediate region of TcdA (TcdA^1102–1847^) to beads and checked for binding to the CROPs (Figure 4C). An immunoblot against GST ensured that equal amounts of beads were used (upper panel). Input and bound toxin fragments were detected through a *C*-terminal histidine epitope (lower panel). The GT-domain, as well as the intermediate region were precipitated by the CROPs, though with less affinity than mutant TcdA^1–1874^, which comprises all toxin domains, except the CROPs. Moreover, binding affinity neither increased by simultaneous incubation of all isolated domains nor by using a fragment consisting of amino acids 543–1847 (Figure S1). Thus, a distinct domain responsible for the observed CROP-interaction could not be identified. This phenomenon was specific to TcdA fragments, since TcdB CROPs immobilized at beads were not capable of precipitating the homologous *N*-terminal TcdB fragments (Figure 5).

While TcdA^1–1874^ was confirmed to be precipitated by TcdA CROPs, neither the isolated glucosyltransferase domain of TcdB (TcdB^1–543^) nor the respective CROP-deletion mutant, TcdB^1–1852^, was detected in the pull-down approach with the CROPs of TcdB (lower right panel).

**Figure 6:**
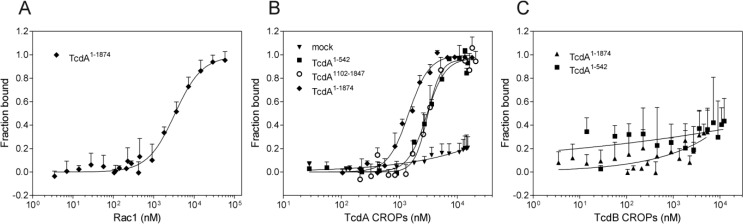
The determination of binding affinities using microscale thermophoresis. Microscale thermophoresis measurements were done to quantify the binding of EGFP-fused TcdA^1–1874^ (♦), TcdA^1–542^ (■) and TcdA^1102–1847^ (○) to unlabeled CROPs. EGFP alone was used as the negative control (mock). The resulting thermophoresis signal was normalized and plotted against the respective CROP concentration. Data were fitted by the Hill slope, and the equilibrium binding constant K_D_ was obtained. Data are presented as means ± SEM (*n* ≥ 4). The R-squared (*R*^2^) reflects the goodness of the respective fit. (**A**) The substrate Rac 1 was used as the positive control for binding to TcdA^1–1874^ (K_D_ = 3.32 ± 0.36 μM); (**B**) Binding of TcdA^1–1874^ to TcdA CROPs resulted in a binding constant of 1.44 ± 0.07 μM (*R*^2^ = 0.98). The affinity of the shorter toxin fragments, TcdA^1–542^ and TcdA^1102–1847^, was less, with K_D_ values of 2.96 ± 0.18 μM (*R*^2^ = 0.96) and 3.06 ± 0.18 μM (*R*^2^ = 0.89); (**C**) In contrast to the CROP domain of TcdA, the TcdB CROPs did not interact with the toxin fragments.

In order to verify the results and to quantify the binding affinities, we took advantage of microscale thermophoresis (MST), a novel and sensitive method that enables the monitoring of the complex formation between fluorescent and non-fluorescent proteins under close-to-native conditions. We therefore titrated EGFP-fused toxin fragments (300 nM) with increasing concentrations of unlabeled TcdA or TcdB CROPs and plotted the resulting thermophoresis signals against the respective CROP concentration (Figure 6).

Since Rac1 is a target substrate of the clostridial toxins, it was used as the positive control and titrated to the fluorescent CROP-deletion mutant, TcdA^1–1874^ (K_D_ = 3.32 ± 0.36 μM). In addition to a standard cytotoxicity assay (Figure S2), the observed dose-dependent binding to Rac1 ensures the correct folding and functionality of the respective EGFP-fused toxin fragments. Similar to the pull-down assays, MST revealed almost identical binding affinities of the glucosyltransferase (TcdA^1–542^) and the intermediate domain (TcdA^1102–1847^) of TcdA with the CROPs, resulting in K_D_ values of 2.96 ± 0.18 μM (■) and 3.06 ± 0.18 μM (○), respectively (Figure 6B). In fact, TcdA^1–1874^, comprising all toxin domains, except the CROPs, bound the TcdA CROP domain with enhanced affinity (K_D_ = 1.44 ± 0.07 μM) compared to the shorter toxin fragments. In contrast, none of the analyzed TcdA mutants showed concentration-dependent binding to the CROPs of homologous TcdB (Figure 6C).

### 2.3. Intramolecular Interactions of TcdA Protect from Its Premature Autoproteolytic Cleavage

**Figure 7:**
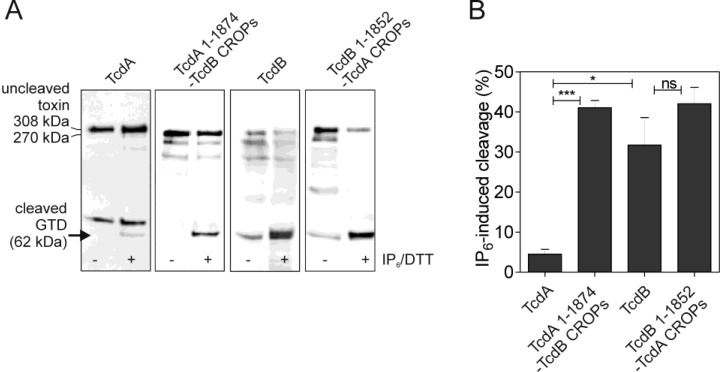
The CROPs of TcdA protect from premature autoproteolytic toxin inactivation. (**A**) Autocatalytic processing of TcdA, TcdB and the chimeras, TcdA^1–1874^-TcdB CROPs and TcdB^1–1852^-TcdA CROPs, respectively. Cleavage was induced by the addition of IP_6_ and DTT at pH 7.0. Specific antibody directed against the glucosyltransferase domain (GTD) of TcdA (indicated by the arrow) was applied in the case of TcdA and TcdA^1–1874^-TcdB CROPs (α-TcdA 542) and against the homologous domain of TcdB in the case of TcdB and TcdB^1–1852^-TcdA CROPs (α-TcdB 543); (**B**) The bar chart shows the densitometrical evaluation of the cleaved glucosyltransferase domain. The cleavage efficacy of TcdA differs significantly from that of chimera TcdA^1–1874^-TcdB CROPs (*** *p* < 0.0001) and TcdB (* *p* = 0.017), respectively; ns = not significant.

**Figure 8:**
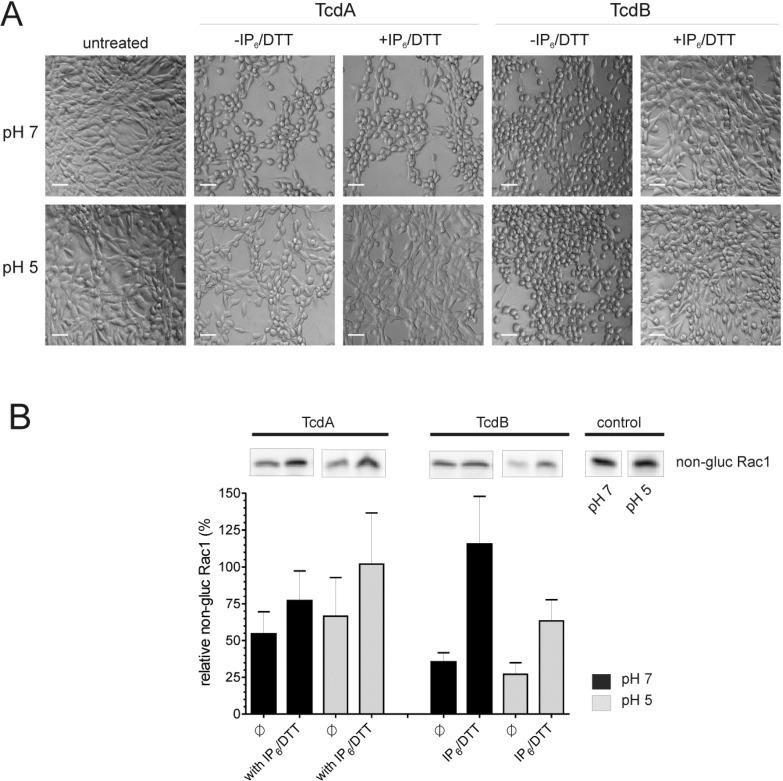
Premature autoproteolysis affects cytotoxicity. (**A**) Representative phase contrast microscopy of 3T3 fibroblasts treated with TcdA or TcdB at pH 7 or pH 5 in the absence or presence of IP_6_/DTT. Cell rounding confirms the successful internalization of the glucosyltransferase domain, which is prohibited by extracellular IP_6_/DTT-induced cleavage. Scale bars represent 50 μm; (**B**) Quantification of relative non-glucosylated Rac1 (in relation to GAPDH) by specific antibody (mean ± SD, *n* = 3). Immunoblots were performed from the samples shown under (**A**); Representative immunoblots are shown in the inserts above the bars.

As indirect proof of intramolecular bonding, IP_6_-induced cleavage of different toxins and chimeras was performed. Based on the previous results, we assume that the close conformation observed at neutral pH for TcdA originates from its CROP domain and sterically hinders the binding of IP_6_ and subsequent activation of the inherent cysteine protease. This might explain the inefficient processing of TcdA at neutral pH, which is observed following the application of the co-factors, IP_6_ and DTT (Figure 7A, first panel). The amounts of the cleaved GT-domain were semi-quantified by western blotting to 4.52% ± 1.17% (Figure 7B). Under the given conditions, the cleavage of a chimera comprising the amino acids 1–1874 of TcdA and the CROPs of TcdB differs significantly, as this mutant is characterized by a ten-fold increase in autocatalysis compared to wild-type TcdA (41.06% ± 1.77%). This behavior reflects an impaired conformation that allows IP_6_-binding and cleavage induction, which shows that toxin structure and inhibition of premature cysteine protease activation is predominantly determined by the CROP domain. Interestingly, this seems to be true only for TcdA, since TcdB was efficiently processed at neutral pH (31.71% ± 6.86%), though being exposed to 100-fold less IP_6_. However, an inhibitory function of the TcdA CROPs only becomes important towards the *N*-terminus of TcdA, rather than the *N*-terminus of TcdB. This conclusion was drawn from a chimera of TcdB^1–1852^ and the TcdA CROP domain, which was as efficiently processed as wild-type TcdB (42.04% ± 4.07%). The linkage between toxin conformation and function was further investigated by a functional assay. As previously shown, extracellular cleavage of the toxins prevents the cytopathic effect of TcdA and TcdB [10]. Rounding of cells treated with TcdA or TcdB, which were previously incubated with IP_6_/DTT, correlates well with the cleavage efficacies described above (Figure 8). Incubation of toxin with IP_6_/DTT at pH 7 hardly affected the potency of TcdA towards 3T3 fibroblasts, due to an ineffective toxin cleavage. In addition to the exchange or deletion of the CROP domain, the protective conformation of TcdA is also impaired during acidification, allowing IP_6_-induced cleavage, and results in the abrogation of the cytopathic effect. In contrast, due to quantitative processing, even at pH 7, TcdB is functionally inactivated in the presence of IP_6_/DTT, and thus, does not significantly affect cell morphology. Cleavage efficiency at pH 5, however, was even less than at pH 7, since the cysteine protease activity itself is reduced under acidic conditions, as could be seen for TcdA^1–1065^ (compare Figure 1A). IP_6_ as an ingredient of dietary fiber is physiologically present in the human large intestine at concentrations reaching 4 mM [14]. Therefore, the necessity for the protection of the cysteine protease by, e.g., intramolecular structures, is obvious. This begs the question of how the TcdB structure is stabilized *in vivo* and protected from premature autoproteolytic cleavage. It is conceivable that external factors, hence intermolecular interactions, rather than an intramolecular structure, stabilize TcdB. This might ensure faster conformational alterations of the toxin and, consequently, a quicker translocation process. Whether both toxins adapt to different niches with TcdA, as the less susceptible molecule, ensuring basic cytotoxicity and TcdB predominantly focusing on efficacy, needs to be elucidated.

## 3. Experimental Section

### 3.1. Antibodies and Reagents

Monoclonal anti-Rac1 antibody recognizing non-glucosylated Rac1 (clone 102, BD PharMingen, Heidelberg, Germany); β-actin antibody (clone AC15, Sigma-Aldrich, Hamburg, Germany); GAPDH antibody, Penta-His antibody (Qiagen, Hamburg, Germany), HRP-conjugated secondary mouse antibody (Rockland, Gilbertsville, PA, USA); *Bacillus megaterium* expression system (MoBiTec, Göttingen, Germany); Inositol hexakisphosphate (Calbiochem/Merck, Darmstadt, Germany).

### 3.2. Expression and Purification of Recombinant Toxins

The *C. difficile* toxins (strain VPI 10463, GenBank Accession No. X51797) were recombinantly expressed in the *B. megaterium* expression system as *C*-terminally His-tagged fusion proteins, unless otherwise noted. Expression and purification was performed following the standard protocol, as described previously [15]. Cloning of recombinant TcdA and TcdB, the isolated TcdA CROPs (TcdA^1875–2710^), the CROP deletion mutants TcdA^1–1874^ and TcdB^1–1852^, as well as fragment TcdA^1–1065^ is described elsewhere [3,16]. The *C*-terminally EGFP-fused constructs, TcdA^1–1874^-EGFP and TcdA^1–542 D285/287N^-EGFP, were generated by mobilization of *tcdA* 1–5622 bp and *tcdA* 1–1626 bp from the host plasmids by the *SpeI* or *SpeI* and *BamHI* restriction sites, respectively, and ligation into the modified *B. megaterium* expression vector pHIS1522 harboring the *egfp* gene (pHIS1522-EGFP). The construct encoding *N*-terminally EGFP-labeled TcdA^1102–1847^ was cloned by amplification of *egfp* from vector pEGFP-C1 (BD Biosciences Clontech, Heidelberg, Germany) and insertion through *BamHI* restriction sites into pQE30 plasmid harboring base pairs *tcdA* 3304-5541. The GST-tagged CROPs of TcdA and TcdB were generated by amplification of base pairs *tcdA* 5623-8130 (sense: 5'-AGCTAGATCTTATAAAATTATTAATGGTAAAC; antisense: 5'-AGTCGGATCCGCCATATATCCCAGGGGCTTTTAC) and *tcdB* 5542-7098 (sense: 5'-AGCTGGATCCCCAGTAAATAATTTGATAA; antisense: 5'-AGCTGAATTCCTTCACTAATCACTAATTG) using the vector, pWH-TcdA, or genomic DNA of *C. difficile* strain VPI 10463 as the template, respectively. The resulting amplicons were digested with *BamHI* and *EcoRI* in the case of TcdB and restriction enzymes *BglII* and *BamHI* in the case of TcdA and were ligated into the prepared pGEX-2T vectors (GE Healthcare, Hamburg, Germany). Expression of the respective GST-tagged gene products occurred in TG1 *E. coli* following IPTG induction.

Purification of the His-tagged toxins and fragments was achieved by Ni^2+^ affinity chromatography. For pull-down experiments, the cleared supernatants of bacterial lysates harboring GST-fused TcdA or TcdB CROPs were incubated with GSH beads overnight at 4 °C in order to obtain the bead-coupled CROP domain. Afterwards, the beads were collected by centrifugation at 1000× *g*, washed three times with 50 mM Tris (pH 8.0), 80 mM NaCl and 0.5% Triton X-100 and used for precipitation experiments.

Chimera TcdB^1–1852^-TcdA^1875–2710^ consists of the TcdA CROP domain fused to the CROP-deletion mutant of TcdB. It was generated by amplification of *tcdA* 5623–8130 using primers 5'-AGCTGGATCCTTTATAAAATTATTAATGGTAAACAC (sense) and 5'-AGTCGCATGCCCGCCATATATCCCAGGGGCTTTTAC (antisense) and ligation into plasmid pHIS1522-TcdB 1–1852 through *BamHI* and *SphI* restriction sites. The reciprocal chimera harboring CROP-truncated TcdA^1–1874^ and the TcdB CROPs was cloned by mobilization of *tcdA* 1–5622 from plasmid pWH-TcdA by *SpeI* restriction and ligation into the respective *SpeI* digested vector, pHIS-TcdB 1848–2366. All constructs are listed in Table S1, showing the location of tags and providing data about linker and additional amino acids as carry-over from the cloning strategy.

### 3.3. Generation of Specific Antibody

For the generation of specific antibodies, either TcdA fragment 1–1065 or the glucosyltransferase-inactive mutant (D286/288N) of TcdB fragment 1–543 were expressed in *B. megaterium* and purified by affinity chromatography and gel extraction. First, immunization of a New Zealand rabbit was performed after the standard protocol with 100 μg of protein followed by a single boost after four weeks. Blood was collected four weeks after boost immunization. The specificity of anti-serum was checked by western blot using the antigens, as well as full-length TcdA and TcdB as positive and negative controls.

### 3.4. Flow Cytometry

Mutant TcdA^1–1874^ was fluorescently labeled with Lightning-Link™ Atto488 as previously described [3], and binding to HT29 cells in dependence of TcdA CROP pre-incubation was analyzed by flow cytometry. Therefore, adherent cells were suspended by Accutase treatment, and 500,000 cells were incubated at 4 °C for 30 min, either directly with 4 nM of fluorescent labeled TcdA^1–1874^ or after pre-incubation for 30 min with non-labeled TcdA CROPs (TcdA^1875–2710^) at 4 °C. Cells were washed twice with ice-cold PBS by centrifugation at 200× *g* for 5 min at 4 °C to eliminate non-bound toxins and finally subjected to flow cytometry (FACScan flow cytometer; Becton Dickinson, Heidelberg, Germany). Ten thousand events were monitored per condition.

### 3.5. Pull-Down

Pull-down experiments were performed in binding buffer (50 mM Tris, 80 mM NaCl, 5 mM KCl, 1 mM CaCl_2_, 1 mM MgCl_2_, 1% (*v*/*v*) NP-40, pH 7.4) at 4 °C for 2 h. As bait, GST-fused TcdA or TcdB CROPs coupled to GSH beads were used, previously blocked for 2 h at 4 °C in 50 M Tris (pH 8.0), 80 mM NaCl, 0.1% NP-40 (*v*/*v*) and 1% BSA (*w*/*v*). Precipitation was done in a total volume of 200 μL with 180 pmol of GST-tagged CROPs and 50 pmol of the respective toxin fragment or H_2_O as the control. Previous to incubation, a sample of each approach was taken as the input control. Following binding, beads were pelleted by centrifugation at 1000× *g* for 5 min at 4 °C and washed three times in 20 mM Tris (pH 8.0), 80 mM NaCl, 0.5% Triton X-100. Finally, toxin fragment-bound beads were resuspended in Laemmli buffer; proteins were denaturized for 10 min at 95 °C, and the soluble fraction was subjected to SDS-PAGE and western blotting.

### 3.6. Microscale Thermophoresis (MST)

Thermophoresis was used to determine the binding affinities between the TcdA or TcdB CROP domain and *N*-terminal TcdA fragments. Therefore, a fixed concentration of 300 nM EGFP-fused TcdA^1–542^, TcdA^1102–1847^ or TcdA^1–1874^, respectively, was titrated with 20 μM to 0.01 μM of TcdA or TcdB CROPs in 20 mM Tris, 50 mM NaCl (pH 7.4). As a positive control, a concentration series of recombinant Rac1 was applied. In order to allow binding, samples were incubated at least 30 min at room temperature followed by centrifugation for 5 min at 15,000× *g* to eliminate potential precipitates. Experiments were performed in standard or hydrophilic capillaries using a NanoTemper Monolith™ NT.115 instrument for green dye fluorescence according to Duhr, Braun and co-workers [17]. Thermophoresis signals for each of the 16 capillaries were monitored, which harbor different ratios of binding partners. The normalized fluorescence at a given time point was plotted against the concentration of unlabeled CROPs. The resulting sigmoidal curves were normalized, and the means ± SEM (*n* ≥ 4) to each data point were determined. Data points were finally fitted by the Hill slope, and K_D_ values were obtained.

### 3.7. IP_6_-Induced Cleavage

Two hundred sixty nanomoles of toxins were incubated for 2 h at 37 °C in 50 mM Hepes (pH range 5.0–8.0, as indicated) and 2 mM dithiothreitol supplemented with 10 μM inositol hexakisphosphate (IP_6_) in the case of TcdB and TcdB^1–1852^-TcdA^1875–2710^ or 1 mM IP_6_ in the case of TcdA, TcdA^1–1065^ and TcdA^1–1874^-TcdB^1848–2366^, respectively. For cytotoxicity assays, samples were taken, neutralized to pH 7.4 and applied to 3T3 fibroblasts at final concentrations of 30 to 900 pM. In order to evaluate cleavage efficacies, cleavage reactions were stopped by boiling at 95 °C for 5 min in Laemmli sample buffer, and after neutralization, samples were subjected to SDS-PAGE and western blot analyses. Antibodies were directed against the *N*-terminus of TcdA (α-TcdA 542) or TcdB (α-TcdB 543), respectively. Densitometrical evaluation is illustrated as a bar diagram showing only IP_6_/DTT-induced cleavage minus the background signal in the absence of the inducers. Data are presented as the means ± SEM (*n* = 3).

### 3.8. Cell Culture and Cytotoxicity Assay

3T3 mouse fibroblasts were cultivated under standard conditions in Dulbecco’s modified Eagle’s medium (DMEM) supplemented with 10% fetal bovine serum (FBS), 100 μM penicillin, 100 μg/mL streptomycin. For cytotoxicity assays, cells were seeded in 24-well chambers and grown for 48 h to sub-confluence. Cleaved and non-cleaved samples were neutralized to pH 7.4, diluted in medium and applied to the cells with final toxin concentrations of 30 pM of TcdB or TcdB^1–1852^-TcdA^1875–2710^ and 900 pM of TcdA or TcdA^1–1874^-TcdB^1848–2366^, respectively. Toxin-induced cell rounding was monitored by light microscopy after 3 h of incubation.

### 3.9. Western Blotting

Protein samples were separated by SDS-PAGE and transferred onto a nitrocellulose membrane. After blocking with 3% (*w*/*v*) BSA and 2% (*w*/*v*) nonfat dry milk in TBST (50 mM Tris HCl pH 7.2, 150 mM NaCl, 0.05% (*v*/*v*) Tween-20), the membrane was incubated overnight with the primary antibody at 4 °C. Following washing with TBST, it was incubated for 45 min at room temperature with horseradish peroxidase-conjugated secondary antibody in TBST. Detection was performed by means of chemiluminescence. Rac1-glucosylation as a direct marker for intracellular toxin action was determined as described earlier [18].

### 3.10. Statistical Analysis

Two-tailed *t*-tests were performed using GraphPad Prism 5.02 (GraphPad Software, San Diego, CA, USA, 2008) to evaluate statistical significance. Significance was set at a *p*-value of <0.05. Data are presented as the mean ± standard error of the mean (SEM).

## 4. Conclusions

The present study describes the CROPs of TcdA to shield toxin conformation. Beyond the role as the main receptor binding domain, the *C*-terminal CROPs interact with the *N*-terminal part of the toxin to prevent premature cleavage and, thus, inactivation of the toxin. In line with this, a lack of interaction between CROPs and the *N*-terminus in TcdB correlated with more efficient autoprocessing. We conclude that the *C. difficile* toxins complement one another, together providing full pathogenic potential under any given condition: TcdA is less potent, but robust against different milieu circumstances, and TcdB, though being more susceptible, ensures efficient cytotoxicity, due to its high potency.

## Supplementary Files

Supplementary File 1 Supplementary Information (PDF, 445 KB)
