# Supplementary material for: The Combined Repetitive Oligopeptides of Clostridium difficile Toxin A Counteract Premature Cleavage of the Glucosyl-Transferase Domain by Stabilizing Protein Conformation
Source: Toxins (Basel). 2014 Jul 22;6(7):2162–76. doi: 10.3390/toxins6072162 (PMC4113749; doi:10.3390/toxins6072162)

Supplementary Information

Figure S1. HT-29 cells were incubated with TcdA$^{1–1874}$ and the fusion protein TcdA1-1874-EGFP for one hour. Both toxins induced comparable cell rounding, indicating that the C-terminal EGFP-Tag does not affect biological function of TcdA$^{1–1874}$.

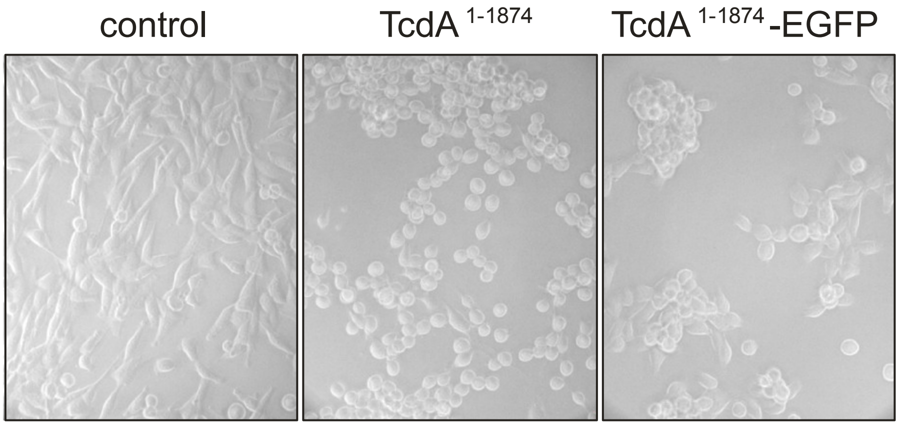

Table S1. Summary of constructs used for this study. Shown are the amino acid sequences of proteins resulting from the cloning strategy.

Construct	Proteins, Tags, Linker	Vector
TcdA$^{1–2710}$	Val-Gln-Thr-Ser-Gly-Ser-[TcdA$^{1–2710}$]-Gly-Ser-**[His]$_6$**	pWH1520
TcdA$^{1–542\ D285/287N}$	Val-Gln-Thr-Ser-[TcdA$^{1–542}$]-Gly-Ser-**[His]$_6$**	pHis1522
TcdA$^{1–1065}$	Gly-Ser-[TcdA$^{1–1065}$]-Gly-Ser-**[His]$_6$**	pWH1520
TcdA$^{1–1874}$	Val-Gln-Thr-Ser-[TcdA$^{1–2710}$]-Thr-Ser-**[His]$_6$**	pWH1520
TcdA$^{1875–2710}$ (CROPs)	Thr-Ser-[TcdA$^{1875–2710}$]-Gly-Ser-**[His]$_6$**	pWH1520
GST-TcdA$^{1875–2710}$ (CROPs)	**[GST]**-[Gly]$_5$-Ser-[TcdA$^{1875–2710}$]-Asp-Pro-Arg-Glu-Phe-Ile-Val-Thr-Asp	pGEX2TGL
GST-TcdB$^{1848–2366}$ (CROPs)	**[GST]**-[Gly]$_5$-Ser-[TcdB$^{1848–2366}$]-Glu-Phe-Ile-Val-Thr-Asp	pGEX2TGL
EGFP-TcdA$^{1102–1847}$	**[His]$_6$**-Gly-Ser-**[EGFP]**-Ser-[TcdA$^{1102–1847}$]	pQE30
EGFP-TcdA$^{1875–2710}$ (CROPs)	Gly-Ser-**[EGFP]**-Ser-[TcdA$^{1102–1847}$]-Gly-Ser-**[His]$_6$**	pWH1520
TcdA$^{1–1874}$-EGFP	Val-Gln-Thr-Ser-[TcdA$^{1–1874}$]-Thr-Ser-**[EGFP]**-Gly-Ser-**[His]$_6$**	pHis1522
TcdA$^{1–542}$-EGFP	Gly-Ser-[TcdA$^{1–542}$]-Thr-Ser-**[EGFP]**-Gly-Ser-**[His]$_6$**	pHis1522
TcdA$^{1–1874}$-TcdB$^{1848–2366}$	[TcdA$^{1–1874}$][TcdB$^{1848–2366}$]-Thr-Ser-**[His]$_6$**	pHis1522
TcdB$^{1–1852}$-TcdA$^{1875–2710}$	[TcdB$^{1–1852}$][TcdA$^{1875–2710}$]-Thr-Ser-**[His]$_6$**	pHis1522
TcdB$^{1848–2366}$ (CROPs)	[TcdB$^{1848–2366}$]-Gly-Ser-**[His]$_6$**	pHis1522
TcdB$^{1–1852}$	[TcdB$^{1–1852}$]-Thr-Ser-**[His]$_6$**	pHis1522
TcdB$^{1–543}$	[TcdB$^{1–543}$]-Gly-Ser-**[His]$_6$**	pHis1522
TcdB$^{1–2366}$	[TcdB$^{1–2366}$]-Gly-Ser-**[His]$_6$**	pHis1522

Figure S2. (**A**) Glutathion-S-Transferase (GST)-Pull down assays showing binding of $TcdA^{1–542}$ (lane 1, "input" and "TcdA-CROPs") or $TcdA^{543–1874}$ (lane 2, "input" and "TcdA-CROPs") and the combination of $TcdA^{1–542}$ and $TcdA^{543–1874}$ (lane 3, "input" and "TcdA-CROPs") to immobilised TcdA-CROPs. Binding of $TcdA^{1–542}$ and $TcdA^{543–1874}$ was compared with positive control $TcdA^{1–1874}$ (lane 4, "input" and "TcdA-CROPs"). Unspecific binding to GST-beads was checked by pull down of $TcdA^{1–542}$ and $TcdA^{543–1874}$ in combination (lane 3, "GST") and of $TcdA^{1–1874}$ (lane 4, "GST"). Combination of $TcdA^{1–542}$ and $TcdA^{543–1874}$ did not increase binding of either domain to TcdA-CROPs; (**B**) GST-Pull down assays showing binding of $TcdA^{1–1101}$ (lane 1, "input" and "CROPs") or $TcdA^{1102–1847}$ (lane 2, "input" and "CROPs") and the combination of $TcdA^{1–1101}$ and $TcdA^{1102–1874}$ (lane 3, "input" and "CROPs") to immobilised TcdA-CROPs. Binding of $TcdA^{1–1101}$ and $TcdA^{1102–1874}$ was compared with positive control $TcdA^{1–1874}$(lane 4, "input" and "CROPs"). Unspecific binding to GST-beads was checked by pull down of $TcdA^{1–1101}$ and $TcdA^{1102–1874}$ in combination (lane 3, "GST") and of $TcdA^{1–1874}$ (lane 4, "GST"). $TcdA^{1–1101}$ did not bind to TcdA-CROPs (lane 1, "CROPs") and did not increase binding of $TcdA^{1102–1874}$ (lane 2, "CROPs") when added in combination of both (lane 3, "CROPs").

A

B

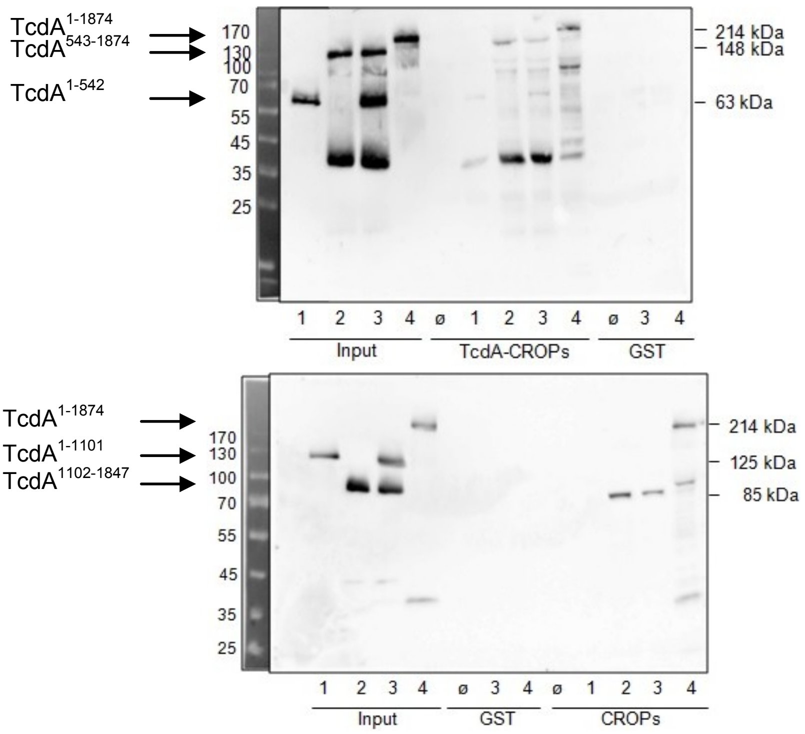

Supplement: Supplementary File 1 — Supplementary Information (PDF, 445 KB) [file toxins-06-02162-s001.pdf]
